# Philadelphia chromosome-positive B-lymphoblastic lymphoma successfully treated with chemotherapy regimen containing imatinib: A rare case report and literature review

**DOI:** 10.1097/MD.0000000000026323

**Published:** 2021-06-11

**Authors:** Xiaoning Li, Weijie Cao, Suping Zhang, Li Li, Yingmei Li, Zhongxing Jiang, Dingming Wan, Jifeng Yu

**Affiliations:** The First Affiliated Hospital of Zhengzhou University, Zhengzhou, China.

**Keywords:** B-lymphoblastic lymphoma, clinical characteristics, literature review, managements, Philadelphia chromosome, prognosis

## Abstract

**Rationale:**

B-lymphoblastic lymphoma (B-LBL) with BCR/ABL mutation (Ph+ B-LBL) is a rare type of cancer in both childhood and adults. Its clinical manifestations are similar to those of other types lymphoma. However, the targeted therapy can substantially improve the outcome of Ph+ B-LBL.

**Patient concerns:**

A 19-year-old male with blood type O, Rh+ was admitted into our hospital on August 14, 2018, due to a recurrent fever and hypocytosis for 6 months.

**Diagnoses:**

Routine blood exam showed pancytopenia. Bone marrow sample flow cytometry (FCM) exam showed abnormal cells were 2.27% of the nucleated cells, and was classified as the abnormal early B-lineage lymphoblastic cells. FISH testing showed the BCR/ABL positive cells were 13.6%. Karyotype analysis showed the 46, XY, t(9;22)(q34;q11). Molecular analysis of BCR/ABL mutation on ABL kinase showed that BCR/ABL T315I mutation. Patient was diagnosed with B-LBL with BCR/ABL mutation (Ph+ B-LBL).

**Interventions:**

The patient was given chemotherapy with VDPI regimen (Vinorelbine, daunorubicin, prednisone, imatinib).

**Outcomes:**

The patient achieved complete remission after 2 courses’ treatment, followed by one course of clarithromycin regimen and another two courses of VDPI regimen. Patient remains in complete remission as of March 10, 2021.

**Lessons:**

In B-LBL, a BCR/ABL mutation can happen in some of these patients. It is important to guide the pathologist to perform appropriate gene mutation detection, in addition to routine Immunohistochemistry test, to ensure an accurate diagnosis and use the targeted agent for treatment. According to the literature and our results, it seems that intensive chemotherapy plus TKI regimen is effective in inducing complete remission, and allo-SCT should be used as a long-term strategy.

## 1 Introduction

Lymphoblastic lymphoma (LBL) is usually of T-cell origin and B-lymphoblastic lymphoma (B-LBL) accounts for about 10% to 15% of cases.^[1]^ The BCR/ABL gene mutation (Ph^+^) is the most common cytogenetic abnormality in chronic myeloid leukemia (CML) as well as in a subset of B-lineage acute lymphoblastic leukemia (B-ALL). However, the BCR/ABL mutation can happen in the B-LBL as the BCR/ABL positive B-LBL (Ph^+^ B-LBL) less frequently, which has been considered as a rare disease. So far, only 6 cases of Ph^+^ B-LBL have been reported in the literature^[2–7]^ as of May 15, 2021, searched by Pub Med with the terms “B-cell lymphoblastic lymphoma” and “philadelphia” or “BCR-ABL”. A summary of the cases reported in literature is listed in Table 1. It often poses a diagnostic challenge and a complete range of immunophenotyping is required for an accurate diagnosis. Besides the involvement of bone marrow, B-LBL may involve extramedullary with renal masses, pleural effusion, skin lesions, tonsillar mass, oropharyngeal mass or multiple lymph nodes involving the cervical, supraclavicular or inguinal regions.^[8]^ Extramedullary presentations made it unique and other differential diagnoses to be considered are DLBCL and Burkitt lymphoma.^[9]^ In the diagnosis of B-LBL, it is important to exclude Burkitt lymphoma. A careful study of the morphology and immunophenotype of the malignant cells are helpful for an accurate diagnosis. The immature lymphoblasts are usually morphologically distinct from mature lymphoid cells.^[10]^ In tissue biopsy, lymphoblasts of B-LBL are typically small to intermediate in size, with a high nuclear-cytoplasmic ratio, fine chromatin and inconspicuous to prominent nucleoli.^[11]^ We describe a case of Ph^+^ B-LBL, who was successfully treated with intensive chemotherapy containing tyrosine kinase inhibitor (TKI) imatinib as the targeted therapy. The patient is disease free for two and half years after diagnosis without allogeneic stem cell transplantation (allo-SCT).

**Table 1:** Summary of case report in literature.

Case	Year	Patient	Immuno-phenotyping	BCR-ABL test method	Treatment	Prognosis	End Status	Total course	References
1	2013	43/Female	IHC: CD34+,focal staining for CD45 and CD20, CD-3, CD5- and CD138-. Flow cytometry: CD19+, CD10+, CD34+ and TdT+, CD45dim and CD22dim.	FISH	Hyper CVAD and MA, dasatinib. prophylactic intrathecal (IT) chemotherapy. Allo-SCT.	CR in 2 months, followed allo-SCT.	Alive after 4 months	∼8 months	Sadrzadeh et al^[3]^
2	2015	27/Male	FCM: CD20+,CD10+,CD19+,TdT+, CD58+,CD38+,CD34-	RT-PCR+FISH	Hyper-CVAD and MA, imatinib	CR in 6 months. Relapsed after 2 months.	Suicided	∼10 months	Zhu et al^[4]^
3	2017	77/Male	IHC CD10+, CD34+, CD43+, BCL2+, TdT+, CD3-, CD5-, and CD20-. FCM:CD10+,CD19+, CD34+, HLA-DR+, CD4dim, and Ig-kappa-	RT-PCR+FISH	Rituximab, hyper-CVAD and dasatinib, intrathecal prophylactic chemotherapy.	CR after initial chemotherapy. Relapsed in 4 years.	Died	∼4 years	Boddu et al^[5]^
4	2018	26/Male	IHC TdT+, CD10+, CD79+, CD34+, PAX5+, and CD20-, CD3-, and CD45-	FISH	Vincristine and dexamethasone +dasatinib and prophylactic intrathecal chemotherapy. Allo-SCT.	CR in 3 months	Alive 4 months after allo-SCT	∼8 months	Alshomar et al^[6]^
5	2019	65/Male	IHC:CD22+,PAX5+,CD10+,TdT+,CD3-,CD20-	FISH	Hyper CVAD/MA+ dasatinib prophylactic intrathecal chemotherapy+ Radiotherapy	CR after 4 cycles treatment	Alive and disease free 5 years after diagnosis.	∼5 years	Takahashi et al^[2]^
6	2021	18/Female	IHC:PAX5+,CD34+,TdT+,CD10+ FCM:CD10+,CD19+,CD34+,CD20- and CD25-.	RT-PCR	Prednisolone+dasatinib;hyper-CVAD +MA +dasatinib. Allo-SCT.	CR after two weeks.	Alive and disease free 4 months after allo-SCT	∼8 months	Yamada et al^[7]^

## 2 Case report

A 19-year-old male with blood type O, Rh+ was admitted into our hospital on August 14, 2018, due to a recurrent fever and hypocytosis for 6 months. Medical, family, and psycho-social history including relevant genetic information has no special issues. Physical examination of his bones, lymph nodes and other systems were unremarkable. Routine blood exam showed that WBC 1.1 × 10^9^/L, Hb 81 g/L, Platelets 8 × 10^9^/L, and no blast cells were found. Bone marrow sample flow cytometry (FCM) exam showed abnormal cells were 2.27% of the nucleated cells, which expressed CD10, CD19, CD34, HLA-DR, CD58, CD123, partially expressed CD13, CD33, no expression of CD38, CD117, CD15, CD20, CD3, CD4, CD8, CD7, CD56, CD11b, CD16, CD71. It was classified as the abnormal early B-lineage lymphoblastic cells. Mature lymphocytes were 17.21%, and the expressions of CD2, CD3, CD5, CD7 were normal. Large granular lymphocyte CD3^+^/CD57^+^ were 9.07%, CD3^+^/CD56^+^ NK-like T cells were 4.51%: CD3^-^/CD56^+^ NK cells were 10.17%. CD19^+^ B cells were 8.86%. No abnormal cells were found in the myeloid and erythriod lineages. Light chain showed multiple clonal expressions. Next-generation sequence analyses were conducted of the leukemia relevant mutations of 22 genes, including FLT3-ITD, NPM1, KIT, CEBPA, DNMT3A, IDH1, IDH2, TET2, EZH2, RUNX1, ASXL1, PHF6, TP53, SF3B1, SRSF2, U2AF1, ZRSR2, NRAS, CBL, SETBP1, ETV6, and JAK2, with the method as described in our previous publication.^[12]^ For the gene length longer than 150 bp, such as FLT3-ITD, alternative RT-PCR method was used for analysis. No related genes mutation was found. PET-CT showed multiple bone lesions. Femoral bone lesions puncture biopsy showed a focal area of infiltration by abnormal lymphoid cells. These cells were small, exhibiting vesicular nuclei and inconspicuous nucleoli. Immunohistochemistry (IHC) staining showed CD20(−), PAX-5(+), CD3(−), CD43(−), CD7(−), CD99(+), TdT (+), CD10 (+), CD33 (−), MPO (−), CD117 (−), CD235a (−), Ki-67 (70%+). The biopsy showed multiple fragments of bony trabeculae diffusely infiltrated by malignant lymphoid cells. Fluorescence in situ hybridization (FISH) analysis was performed on the bone lesions biopsy to exclude Burkitt lymphoma, by studying the status of the MYC gene. Using a break-apart probe, the FISH analysis showed no evidence of MYC gene rearrangement. FISH testing showed the BCR/ABL positive cells were 13.6%. Karyotype analysis showed the 46, XY, t(9;22)(q34;q11). Molecular analysis of BCR/ABL mutation on ABL kinase showed that BCR/ABL T315I mutation.

In view of the morphological features of the malignant cells and strong positivity for TdT, combining with the clinical manifestation and laboratory testing results especially the biopsy results, the patient was diagnosed with B-LBL with BCR/ABL mutation. The patient was given chemotherapy with VDPI regimen (Vinorelbine, daunorubicin, prednisone, imatinib) and achieved complete remission (CR) after 2 courses’ treatment, followed by one course of clarithromycin regimen and another two courses of VDPI regimen. Patient also received prophylactic intrathecal chemotherapy once for each treatment course. During the chemotherapy, the patient had a mild nausea and moderate pancytopenia. No other severe adverse reactions were found. Patient remains in CR as of January 10, 2021.

## 3 Discussion

The diagnosis of B-LBL has mostly depended on the characteristics of B-lymphoblast leukemia/lymphoma by IHC and a complete range of immunopheotyping, in addition to the molecular finding of the BCR/ABL gene mutation. In most cases of B-LBL, immunophenotyping shows positivity for CD19, CD79a, CD10, PAX5 and TdT, and CD20 is usually negative.^[11]^ In our case, the patients had the PAX5, TdT and CD10 expression, in addition to the BCR/ABL gene mutation. For most B-LBL patients, BCR/ABL-like had been reported in different studies. Only 6 cases B-LBL patients with the BCR/ABL mutation had been reported so far, due to the rarity of disease. However, due to the uncommon of BCR/ABL gene mutation in B-LBL, some patients may not be diagnosed due to the lack of gene mutation laboratory test. So the real incidence of Ph+ B-LBL may be underestimated.

At present, the treatment of Ph^+^ LBL has not been standardized with consensus in the hematology/oncology field. Since multiple targeted tyrosine kinase inhibitors have been developed to specifically target the BCR/ABL mutation in leukemia/lymphoma patients, diagnosis of the subtype of the leukemia/lymphoma with the BCR/ABL mutation will be very important in regarding the patient's treatment. Although the BCR/ABL mutation B-LBL is rare, it's important to diagnose these patients in the early stage for specific therapeutic regimen and the long-term survival benefit. TKI combined with allo-SCT has demonstrated with improved prognosis for this rare disease. As for the chemotherapy regimen, it seems like that the regimen according to acute lymphocytic leukemia (ALL) protocol is preferred. In addition, it has been revealed that achieving early molecular response is associated with low risk of relapse.^[13]^ Among the 6 cases reported, 5 of the patients were treated with hyper-CVAD/MA regimen (cyclophosphamide, vincristine, doxorubicin and dexamethasone, alternating with high-dose methotrexate and cytarabine) combining with the TKI dasatinib. Three of 6 patients underwent the allo-SCT.

As for the prognosis, among these all 6 cases had been reported, 4 of the patients including 3 who underwent allo-SCT were alive at the end of follow-ups. Our case was treated with systemic intensive chemotherapy containing the TKI imatinib as targeted therapy and achieved very good results. Patient remains in CR condition for two and half years without SCT. According to the literature and our results, it seems that intensive chemotherapy plus TKI regimen is effective in inducing complete remission, and allo-SCT should be used as a long-term strategy. However, the adequate treatment strategy needs to be further defined in a large number of cases in the future.

## 4 Conclusion

Our case illustrates that in B-LBL, a BCR/ABL mutation can happen in some of these patients. Definitely, Ph+ B-LBL is a rare entity. However, it may get underdiagnosed due to the failure to identify the gene translocation. It is important to guide the pathologist to perform appropriate gene mutation detection by checking for Philadelphia chromosomes in all cases of B-LBL routinely, in addition to IHC test, to ensure an accurate diagnosis and use the targeted agent for treatment.

## Author contributions

**Conceptualization:** Xiaoning Li, Weijie Cao, Jifeng Yu.

**Data curation:** Xiaoning Li, Weijie Cao, Suping Zhang, Li Li, Yingmei Li, Jifeng Yu.

**Funding acquisition:** Yingmei Li, Jifeng Yu.

**Investigation:** Dingming Wan, Jifeng Yu.

**Resources:** Yingmei Li, Zhongxing Jiang, Dingming Wan.

**Supervision:** Jifeng Yu.

**Writing – original draft:** Jifeng Yu.

**Writing – review & editing:** Jifeng Yu.
